# A Faecal Contamination Index for interpreting heterogeneous diarrhoea impacts of water, sanitation and hygiene interventions and overall, regional and country estimates of community sanitation coverage with a focus on low- and middle-income countries

**DOI:** 10.1016/j.ijheh.2018.11.005

**Published:** 2019-03

**Authors:** Jennyfer Wolf, Richard Johnston, Paul R. Hunter, Bruce Gordon, Kate Medlicott, Annette Prüss-Ustün

**Affiliations:** a Department of Public Health, Environment and Social Determinants of Health, World Health Organization, 20 Avenue Appia, Geneva, Switzerland; b The Norwich School of Medicine, University of East Anglia, Norwich, UK; c Department of Environmental Health, Tshwane University of Technology, Pretoria, South Africa

**Keywords:** Sanitation, Diarrhea, Estimates, Fecal contamination, Sanitation coverage, Community, WASH, water sanitation and hygiene, FAECI, Faecal Contamination Index, PSU, primary sampling unit, SDG, Sustainable Development Goal, JMP, WHO/UNICEF Joint Monitoring Programme for Water Supply Sanitation and Hygiene, DHS, Demographic and Health Survey, MICS, Multiple Indicator Cluster Survey

## Abstract

**Objectives:**

The impact on diarrhoea of sanitation interventions has been heterogeneous. We hypothesize that this is due to the level of prevailing faecal environmental contamination and propose a Faecal Contamination Index (FAECI) of selected WASH indicators (*objective 1*). Additionally, we provide estimates of the proportion of the population living in communities above certain sanitation coverage levels (*objective 2*).

**Methods:**

*Objective 1*: Faecal contamination post-intervention was estimated from WASH intervention reports. WASH indicators composing the FAECI included eight water, sanitation and hygiene practice indicators, which were selected for their relevance for health and data availability at study- and country-level. The association between the estimated level of faecal environmental contamination and diarrhoea was examined using meta-regression. *Objective 2*: A literature search was conducted to identify health-relevant community sanitation coverage thresholds. To estimate total community coverage with basic sanitation in low- and middle-income countries, at relevant thresholds, household surveys with data available at primary sampling unit (PSU)-level were analysed according to the identified thresholds, at country-, regional- and overall level.

**Results:**

*Objective 1*: We found a non-linear association between estimated environmental faecal contamination and sanitation interventions’ impact on diarrhoeal disease. Diarrhoea reductions were highest at lower faecal contamination levels, and no diarrhoea reduction was found when contamination increased above a certain level. *Objective 2*: Around 45% of the population lives in communities with more than 75% of coverage with basic sanitation and 24% of the population lives in communities above 95% coverage, respectively.

**Conclusions:**

High prevailing faecal contamination might explain interventions' poor effectiveness in reducing diarrhoea. The here proposed Faecal Contamination Index is a first attempt to estimate the level of faecal contamination in communities. Much of the world's population currently lives in faecally contaminated environments as indicated by low community sanitation coverage.

## Highlights

• An index for estimating faecal contamination in the community is proposed that combines a broad set of WASH indicators.
• Faecal community contamination is an important determinant of a sanitation intervention's effectiveness to reduce diarrhoea.
• Above a certain level of faecal contamination, interventions are less likely to lead to reductions of diarrhoea.
• Less than half of the global population lives in communities in which coverage with basic sanitation services exceeds 75%.

## 1 Introduction

Drinking water, sanitation and hygiene (WASH) interventions may amongst others provide community water access or household water connections, source or point-of-use water quality improvements, on-site sanitation or sewer connections, handwashing promotion or general hygiene education (Fewtrell et al., 2005). These interventions have been shown to improve health (Freeman et al., 2017; Prüss-Ustün et al., 2008; Wolf et al., 2018a) and to have many non-health benefits such as improvements in equity, dignity, safety, time savings and cognitive development, educational attainment and national economic and overall development (Bapat and Agarwal, 2003; Bartram and Cairncross, 2010; Sclar et al., 2017). Access to safely managed sanitation services, i.e. toilets from which excreta are treated and disposed of safely, is therefore part of Sustainable Development Goal (SDG) 6 (United Nations, 2018).

Recent large, well-designed and well-conducted WASH trials and intervention studies, which included sanitation improvements with high intervention fidelity, did however not yield expected effects on diarrhoeal and nutritional outcomes (Humphrey, 2018; Luby et al., 2018; Null et al., 2018; Reese et al., 2018), which motivated ongoing discussions among researchers, practitioners and funders (Arnold et al., 2018; Coffey and Spears, 2018; Cumming and Curtis, 2018; Rosenboom and Ban, 2018a, 2018b).

**Fig. 1:**
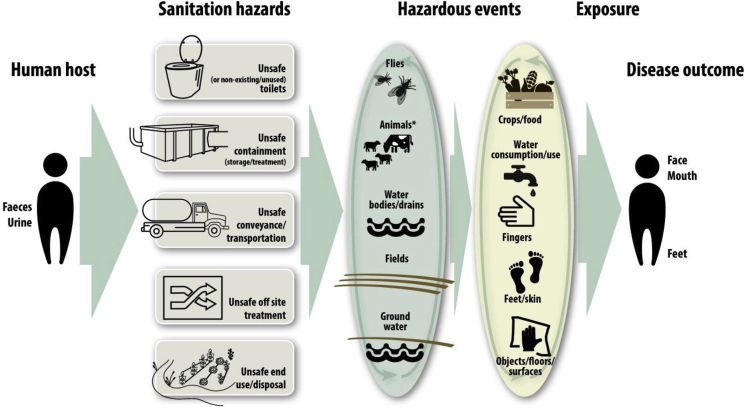
**The health impacts from unsafe sanitation through various transmission pathways (Figure taken from (**WHO, 2018a**)),** * Refers to animals as mechanical vectors. Transmission of animal excreta-related pathogens to human hosts is not represented in this diagram.

It has long been argued that the - measurable - impact of WASH interventions on diarrhoea depends on the number and type of faecal pathogens that people are exposed to through a variety of transmission pathways in their homes and communities (Briscoe, 1984; VanDerslice and Briscoe, 1995) (Fig. 1). In communities with very high levels of faecal contamination, an intervention that successfully reduces faecal exposure through one exposure pathway might still translate in no or only slight diarrhoea reduction, in particular if other important pathways remain (VanDerslice and Briscoe, 1995). The SaniPath study, for example, has provided insights into pathways of exposure to faecal contamination, has revealed high levels of faecal environmental contamination in low-income settings and identified the consumption of contaminated food as the main fecal exposure pathway for children (Robb et al., 2017). Another example is faecally contaminated drinking water, which can result from inadequate sanitation and hygiene and which has been shown to be a frequent problem even in so-called “improved” drinking water sources (Bain et al., 2014). Public and occupational settings might offer important additional exposure routes through for example contaminated soil and open drains (Antwi-Agyei et al., 2016; Berendes et al., 2018).

There is consistent and considerable evidence that entire communities benefit from sanitation improvements in individual households, e.g., (Andres et al., 2014; Fuller et al., 2016; Garn et al., 2018; Jung et al., 2017a; Larsen et al., 2017; VanDerslice and Briscoe, 1995). The health impacts from sanitation might even primarily result from protective effects on the community rather than from direct benefits to individual households (Andres et al., 2014; Fuller and Eisenberg, 2016; Harris et al., 2017). High community coverage of sanitation might be especially important for densely-populated areas with frequent person-to-person contact and little free space (Berendes et al., 2017).

This paper is motivated by two main objectives: The first is to estimate faecal environmental contamination post-intervention from sanitation intervention reports and to use these estimates to explain heterogeneous impacts on diarrhoeal disease. For this purpose we propose for the first time a Faecal Contamination Index (FAECI) of selected WASH indicators. The second objective is to provide country, regional and overall estimates of the proportion of the population living in communities above a certain level of coverage with basic sanitation services with a focus on low- and middle-income countries to complete data on faecal environmental contamination in general and on WASH indicators used for the FAECI.

## 2 Methods

### 2.1 Estimation of faecal environmental contamination using a composite index and assessing the association between faecal contamination and the impact of sanitation interventions on diarrhoeal disease

#### 2.1.1 Construction of the Faecal Contamination Index (FAECI)

A set of WASH indicators was selected to develop a Faecal Contamination Index (FAECI), on the basis of biological plausibility, demonstrated association with diarrhoeal disease (Ram et al., 2014; Wolf et al., 2018a), data availability at country-level and frequent reporting in research trials. Since poor sanitation is a primary cause of faecal environmental contamination, half of the eight indicators relate to sanitation practices. Two indicators were selected for drinking water and two for hand hygiene. Seven of the indicators refer to the proportion of the population (at the scale of the intervention) that:

1. practice open defecation or unsafe disposal of child faeces (S1),
2. use or have access to basic sanitation services, i.e., improved sanitation facilities that are not shared between two or more households (S2),
3. use or have access to safely managed sanitation services, i.e., basic sanitation services that ensure safe disposal or safe transport and treatment of excreta (S3),
4. use or have access to basic drinking water services, i.e., water from improved sources that require no more than 30 min to collect water from (W1),
5. use or have access to safely managed water services, i.e., improved sources accessible on premises, which provide water free from contamination and available when needed (W2),
6. have access to basic handwashing facilities, i.e., handwashing facilities with soap and water on premises (H1),
7. wash hands with soap after potential faecal contact (H2).

**Fig. 2:**
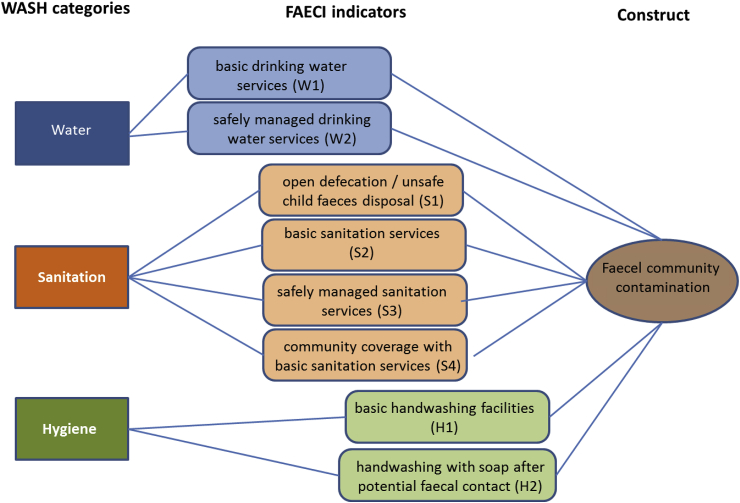
WASH indicators for assessing faecal environmental contamination.

An eighth indicator (S4) refers to community sanitation coverage, i.e. the proportion of the population within a community that uses or has access to basic sanitation services. The eight indicators are shown in Fig. 2.

Indicators S2, S3, S4, W1 and W2 refer to either use of or access to services dependent on the information available from the study report. S1 and H2 are indicators of behaviours whereas H1 assesses access to services. More information on the indicators and scoring of interventions is provided in Table 1. We subjectively chose 75% and 50% as the cut-off values for most indicators as a simplified approach and in order to get a good distribution of scores. S1 (open defection/unsafe child faeces disposal), S3 (safely managed sanitation services) and W1 (basic drinking water services) received more stringent cut-off values because we assumed high potential faecal contamination of the environment from open defecation and unsafely managed sanitation services (WHO, 2018a). Most of the world's population is now covered with improved drinking water sources (WHO and UNICEF, 2017), which are however often faecally contaminated (Bain et al., 2014). A rather high cut-off value for W1 ensured a better distribution of scores. Improved drinking-water and sanitation facilities are defined following the JMP (WHO and UNICEF, 2017).

**Table 1:** Indicators for assessing faecal environmental contamination: scoring and further information.

WASH category	Scoring [number of points]^a^	Notes
Sanitation	(percentages usually relate to percentage of population)	
S1. Open defecation (OD)/unsafe child faeces disposal	[0] <5% OD, unsafe child faeces disposal [1] 5%–10% [2] >10% OD	If open defecation was not directly reported in the study, we used information on sanitation coverage and reported use of toilets. If child faeces disposal was not reported in the study we assumed that there was no unsafe child faeces disposal.
S2. Basic sanitation services	[0] ≥75% use or access to basic sanitation services [1] ≥50%-<75% [2] <50%	This indicator usually measures the proportion of the study population that has been provided with or that uses the intervention facilities. It also reflects functionality of services if such information is available.
S3. Safely managed sanitation services	[0] <5% use or access to unsafely managed sanitation (such as open drains/flush on street) [1] 5%–10% [2] >10%	Due to data constraints, this indicator assesses evidence against safe management of sanitation facilities (such as open drains, overflowing toilets, unimproved facilities). Sanitation facilities were assumed to be safely managed when the three following conditions were met: there was no indication of unsafe management, intervention facilities were basic sanitation services and the majority of the study group was covered with these facilities. The same cut-offs for scoring as for OD/unsafe child faeces disposal are applied as we assumed high potential faecel contamination from facilities with evidence against safe management.
S4. Community coverage with basic sanitation services	[0] ≥75% community coverage with basic sanitation services [1] ≥60%-<75% [2] <60%	This indicator assesses coverage with the intervention facilities of the whole community. It is referring to use of sanitation if such information is available.
Drinking water		
W1. Basic drinking water services	[0] ≥ 90% use or access to a basic drinking water service [1] ≥75%-<90%, or ≥ 90% but evidence for household water storage [2] <75%	Basic drinking water services are defined as improved drinking water sources from which water is available in <30min round-trip (WHO and UNICEF, 2017). As the time to collect water is often not reported in intervention studies, we use here the proportion of improved drinking water sources without the information on distance. If information on this indicator was not available from the study, it was replaced with country-representative data for the respective country, setting and year (WHO and UNICEF, undated).
W2. Safely managed drinking water services	[0] ≥75% use or access to safely managed drinking water services [1] ≥50%-<75% [2] <50%	Safely managed drinking water services include water that is accessible on premises, available when needed and free from contamination. As the continuity of supply is often not reported in sanitation interventions studies, safely managed drinking water services are operationalized as the proportion of improved drinking water supplies on premises that is free from contamination. If information on this indicator is not available from the respective study, it has been replaced with country-representative data for the respective country, setting and year (WHO and UNICEF, undated).
Hygiene		
H1. Basic handwashing facilities	[0] ≥ 75% access to basic handwashing facilities [1] ≥50% - <75% [2] <50%	This indicator measures access to a handwashing facility on premises that is equipped with water and soap. If presence of basic handwashing facility was not given, the value was replaced with country representative JMP data for the respective country, setting and year (WHO and UNICEF, undated) or from an analysis modelling the presence of basic handwashing facilities at country level (Wolf et al., 2018b).
H2. Handwashing with soap after potential faecal contact	[0] ≥75% wash hands with soap after potential faecal contact [1] ≥50% - <75% [2] <50%	This indicator reflects observed handwashing with soap (HWWS) after potential faecal contact. If observed HWWS is not reported, the indicator was approximated by reported HWWS or by the proportion of basic handwashing facilities (Wolf et al., 2018b)

a A high score represents high estimated faecal contamination.

The first six indicators are assessed regularly (S1: only open defecation) through nationally-representative household surveys and are compiled and reported globally by the JMP every two years (WHO and UNICEF, undated). Child faeces are considered safely disposed of if the child him/herself uses a toilet or latrine, or if another person puts or rinses the child's faeces into a toilet or latrine (Water and Sanitation Program, 2015). The proportion of the population using safely managed drinking water services, safely managed sanitation services and a handwashing facility with soap and water are global indicators of the Sustainable Development Goals (SDG indicator 6.1.1 and 6.2.1) (United Nations, n.d.). The population using basic drinking water and sanitation services, and with access to handwashing facilities also contribute to monitoring of SDG targets on poverty (indicator 1.4.1). Structured observation of handwashing with soap after potential faecal contact is considered the most reliable way to measure actual hand hygiene behaviour (Luby et al., 2011; Ram, 2013) and has been estimated by country for the year 2015 based on the JMP global database and nationally-representative household surveys (Wolf et al., 2018b) (H2). The proportion of community sanitation coverage (S4) has recently been examined as an important determinant for reducing diseases and other health conditions related to basic sanitation services (Jung et al., 2017a; Larsen et al., 2017; Wolf et al., 2018a). Community sanitation coverage represents a distinct pathway for faecal contamination from household-level sanitation (S2). Estimates of community sanitation coverage are now being presented in this work at national, regional and overall level for low- and middle-income countries (Objective 2).

#### 2.1.2 Estimating faecal environmental contamination in published sanitation intervention studies using the FAECI

The potential level of faecal environmental contamination using the FAECI was estimated in sanitation interventions identified in a recent systematic review (Wolf et al., 2018a). This review included sanitation interventions that reported the effect on diarrhoea morbidity of any improvements in sanitation access or use conducted at the level of the household or community. Interventions in non-household settings such as schools, healthcare facilities, or workplaces were not included. Interventions needed to be tested against a control group that did not receive the respective intervention(s) or that received a control or placebo intervention. Eligible study designs included randomized controlled trials, quasi-randomized and non-randomized controlled trials when baseline data on the main outcome were available, case–control and cohort studies when they were related to a clearly specified intervention and studies using time-series designs (Wolf et al., 2018a). Additionally to the interventions identified from the systematic review (Wolf et al., 2018a), more recently conducted sanitation and WASH trials and intervention studies based on the same inclusion criteria were included (Table 3). When a study made sanitation interventions in both a combined WASH and an exclusive sanitation intervention arm, we selected the exclusive sanitation arm. Each study was evaluated on each of the eight WASH indicators both in the intervention (after the intervention) and the control group. Each indicator was given a score depending on the level of (assumed) faecal contamination for the respective indicator, between zero and two points. Missing information on hygiene or drinking water was extrapolated for the respective country, year and region (urban or rural) from national mean values (WHO and UNICEF, undated; Wolf et al., 2018b). Two raters (AP and JW) independently assessed each study. Discrepancies were discussed and – in case no agreement could be reached – a third person consulted (RJ). The scores of all indicators were added up giving equal weight to each indicator. A large FAECI score represents high estimated faecal contamination in the community or the environment.

**Table 2:** Search terms for literature search.

	Search terms
Construct 1	(prevalence[tw] OR incidence[tw] OR risk[tw] OR exposure[tw] OR exposed[tw] OR outcome[tw] OR epidemiology[tw] OR epidemiological[tw] OR impact[tw] OR effect[tw] OR evaluation[tw] OR odds[tw])
Boolean operator	AND
Construct 2	(neighbourhood[tiab] OR neighbourhoods[tiab] OR neighborhood[tiab] OR neighborhoods[tiab] OR village[tiab] OR villages[tiab] OR community[tiab] OR communities[tiab] OR “herd protection”[tiab] OR “herd protective”[tiab] OR coverage[tiab])
Boolean operator	AND
Construct 3	(toilet*[tiab] OR latrine*[tiab] OR pit[tiab] OR pits[tiab] OR sanita*[ tiab] OR feces[tiab] OR faeces[tiab] OR fecal[tiab] OR faecal[tiab] OR excre*[tiab] OR sewage[tiab] OR sewer*[tiab] OR sewerage[tiab] OR open defecation"[tiab] OR Toilet Facilities"[MeSH] OR Toilet Training"[MeSH] OR Sanitation[MeSH] OR Feces[MeSH] OR Sewage[MeSH])

**Table 3:** Included sanitation and combined WASH interventions.

reference	country	setting	intervention type	improvement of access versus sanitation promotion*	RR (lcl, ucl), p-value#	FAECI post-intervention (intervention group)ǂ
Aziz et al. (1990)	Bangladesh	rural	improved household sanitation plus hygiene education and improved water supply	sanitation access	**0.74 (0.69, 0.80), p<0.01**	9
Briceño et al. (2015)	Tanzania	rural	improved household sanitation	sanitation promotion	0.99 (0.75, 1.30)	15
Clasen et al. (2014)	India	rural	improved household sanitation	sanitation access	0.97 (0.83, 1.12)	13
Garrett et al. (2008)	Kenya	rural	improved household sanitation plus hygiene education and improved water supply	sanitation promotion	**0.31 (0.23, 0.41)**	16
Godfrey et al. (2014)	Mozambique	rural	improved household sanitation	sanitation promotion	0.54 (0.29, 1.01)	15
Humphrey (2018)	Zimbabwe	rural	improved household sanitation plus hygiene education and improved water supply	sanitation access	1.18 (0.87, 1.61), p = 0.3	10
Khush and London (2009)	India	rural	improved household sanitation plus hygiene education and improved water supply	sanitation promotion	1.00 (0.43, 2.32)	12
Klasen et al. (2012)	Yemen	urban	sewer intervention	sanitation access	0.81 (0.35, 1.90)	7
Luby et al. (2018)	Bangladesh	rural	improved household sanitation	sanitation access	**0.61 (0.46, 0.81)**	11
Messou et al. (1997)	Ivory Coast	rural	improved household sanitation plus hygiene education and improved water supply	sanitation access	**0.71 (0.56, 0.91)**	9
Moraes et al. (2003)	Brazil	urban	sewer intervention	sanitation access	**0.31 (0.28, 0.34), p<0.0001**	3
Null et al. (2018)	Kenya	rural	improved household sanitation	sanitation access	0.99 (0.88, 1.1)	11
Patil et al. (2014)	India	rural	improved household sanitation	sanitation promotion	0.97 (0.78, 1.22)	15
Pickering et al. (2015)	Mali	rural	improved household sanitation	sanitation promotion	0.93 (0.76, 1.14), p = 0.5	15
Pradhan and Rawlings (2002)	Nicaragua	urban	sewer intervention	sanitation access	0.43 (0.11, 1.71)	3
Reese et al. (2018)	India	rural	improved household sanitation plus hygiene education and improved water supply	sanitation access	0.98 (0.78, 1.23), p = 0.9	9
Walker et al. (1999)	Honduras	rural	improved household sanitation	sanitation access	**0.65 (0.47, 0.90)**	6

RR: relative risk, lcl: lower 95% confidence limit, ucl: upper 95% confidence limit, improved household sanitation include any improvements to sanitation facilities at household level, sewer interventions provide households with connections to the public sewer system, relative risks in bold indicate results significant at p < 0.05 (confidence limits do not include 1), * “sanitation access” means that the intervention provided (most of) the intervention hardware, it does not exclude sanitation promotion, “sanitation promotion” means that the intervention or project promoted the building of sanitation facilities but did not build or provide them to households, # p-value only added when it could be extracted alongside the relative risk from the publication of the respective intervention study, ^**ǂ**^ the FAECI score can reach a maximum of 16, a high FAECI represents high estimated faecal environmental contamination.

#### 2.1.3 Analysis of the association between estimated faecal contamination (using the FAECI) and the effectiveness of sanitation interventions on diarrhoeal disease

Relative risk estimates for diarrhoeal disease prevalence or incidence were extracted from a recent systematic review (Wolf et al., 2018a) or directly from the sanitation interventions if not included in the review. As odds ratios can overstate the estimated intervention effect (Davies et al., 1998), these were converted to risk ratios using the control group risk as given in the respective paper (Higgins and Green, 2011; Zhang and Kai, 1998). Meta-regression analysis was used to assess the association between the relative risk estimates as outcome and the FAECI as continuous explanatory variable. To assess a hypothesized non-linear relationship between the relative risk estimates and the FAECI, a squared term of the FAECI was introduced in the meta-regression model. Because of the limited number of studies (n = 17) we decided a-priori that no further polynomials would be tested. We however examined the effect of urban versus rural setting (binary variable), combined WASH versus exclusive sanitation interventions (binary variable) and the size of the difference between the FAECI scores in intervention and control group (“delta FAECI”, continuous variable). We also examined the association between the relative risk estimates and the delta FAECI, i.e., the difference between the FAECI score in the intervention and the control group, as single predictive variable – in the meta-regression model. Data analysis was performed using Stata 14 (StataCorp, 2015). Studentized deleted residuals were examined for each study visually departing from the overall trend to examine potential outliers, which can distort results and conclusions from any meta-analytic model (Viechtbauer and Cheung, 2010).

### 2.2 Estimation of country, regional and total proportions of the population from low- and middle-income countries living in communities in which basic sanitation coverage exceeds a defined threshold

#### 2.2.1 Literature search to establish health-relevant community sanitation coverage thresholds

We searched PubMed (in January 2018) combining both MeSH terms and keywords in order to identify studies assessing health effects from improving sanitation services at various community coverage levels (Table 2). We restricted the search to the last 10 years and excluded animal studies (used the humans filter in PubMed).

Sanitation coverage in a community is defined here as the proportion of people in a community that use basic sanitation services. A community has been defined as a “group of people with diverse characteristics who are linked by social ties, share common perspectives, and engage in joint action in geographical locations or settings” (MacQueen et al., 2001). Communities in the current analysis are represented by the survey clusters, or primary sampling units (PSUs), which usually consist of geographic areas that group together approximately 100 households (ICF International, 2012).

#### 2.2.2 Data extraction from national household surveys

We searched the JMP household survey data repository in July 2017 for relevant microdata at PSU-level from Demographic Health Surveys (DHS) (The DHS Program, undated) or Multiple Indicator Cluster Surveys (MICS) (UNICEF, undated), for 195 countries and territories collected since 1998 and containing information on household sanitation access and on data sampling (i.e. specifying the PSU and household weights). When several household surveys with relevant microdata at PSU-level were available we only extracted data from the most recent survey. Extracted data was substituted, if available, by harmonized files prepared by the JMP after specific country consultations. For five countries (Nicaragua, Ecuador, Brazil, China, and Sri Lanka) non-DHS/non-MICS survey data were used as they contained the relevant data and DHS or MICS survey data were not available or had been collected a longer time ago (Nicaragua: ENDESA (Encuesta Nicaragüense de Demografía y Salud) 2011 (UNFPA Nicaragua, 2016); Ecuador: ENEMDU (Encuesta Nacional de Empleo, Desempleo y Subempleo) 2016 (Instituto Nacional de Estadística y censos, 2016); Brazil: PNAD (Pesquisa Nacional por Amostra de Domicílios) 2015 (Instituto Brasileiro de Geografia e Estatística, undated); China: SAGE (WHO Study on global AGEing and adult health) 2008 (WHO, n.d.); Sri Lanka: WHS (World Health Survey) 2003 (World Bank, 2013). A pooled dataset was created combining all countries for which microdata at PSU-level were available. Variables indicating proportion of households using improved sanitation facilities and basic sanitation services were calculated following the JMP definitions (WHO and UNICEF, 2017). Population weights were used in the analysis to estimate the proportion of people living in communities with defined sanitation coverage and to account for the fact that not all households were selected with equal probability into the survey sample. Population weights were calculated by multiplying the household weight as given in the surveys with the number of de jure household members (usual household members, i.e. not only those present at the time of the survey).

#### 2.2.3 Analysis of the population living in communities above a defined threshold of sanitation coverage

Data were analysed taking account of cluster sampling in survey data collection (using the svy-command in Stata). To calculate the percentage of the population living in communities above a defined threshold of coverage with basic sanitation services by country, two analysis steps were performed: first, the proportion of the population within the PSU using basic sanitation services was calculated. Second, the mean proportion of the national population living in clusters having at least a certain basic sanitation coverage level was calculated weighted by the mean of population weights by PSU.

Confidence intervals at country-level were generated using the standard error of the mean at country-level (Statalist - archive, 2009). Regional and global estimates and their 95% confidence intervals were derived using country population figures from the United Nations Population Division for the year 2016 (2017 revision) (United Nations Population Division, undated). Standard errors at regional and global levels were estimated with an approach using the delta method ((De Onis et al., 2004), formula in Appendix A – Main (A.1)). Data analysis was performed in Stata 14 (StataCorp, 2015).

We are following guidelines for accurate and transparent health estimates reporting (“GATHER: Guidelines for Accurate and Transparent Health Estimates Reporting,” n.d.; Stevens et al., 2016) and have included a GATHER-checklist as an Appendix (Appendix B – Gather Checklist). Data analysis code can be obtained from the corresponding author upon request. Ethical clearance was not needed for this work as this study did not involve any human or animal participation or personal data. All data was anonymised and informed consent of the WASH intervention studies was obtained at the time of original data collection.

## 3 Results

### 3.1 Estimation of faecal environmental contamination using a composite index and assessing the association between faecal contamination and the impact of sanitation interventions on diarrhoeal disease

#### 3.1.1 Estimating faecal environmental contamination in published sanitation intervention studies using the FAECI

A total of 17 intervention studies were included (Table 3). Of those, six were combined WASH interventions including a sanitation component, and 11 were exclusive sanitation interventions. Fourteen interventions improved household sanitation and three interventions provided sewer connections. Table 3 shows the FAECI scores post-intervention in the intervention group. The scores for all eight indicators for all 17 studies both in the intervention and control groups are included in Appendix C - Faecal contamination score of sanitation interventions.

**Fig. 3:**
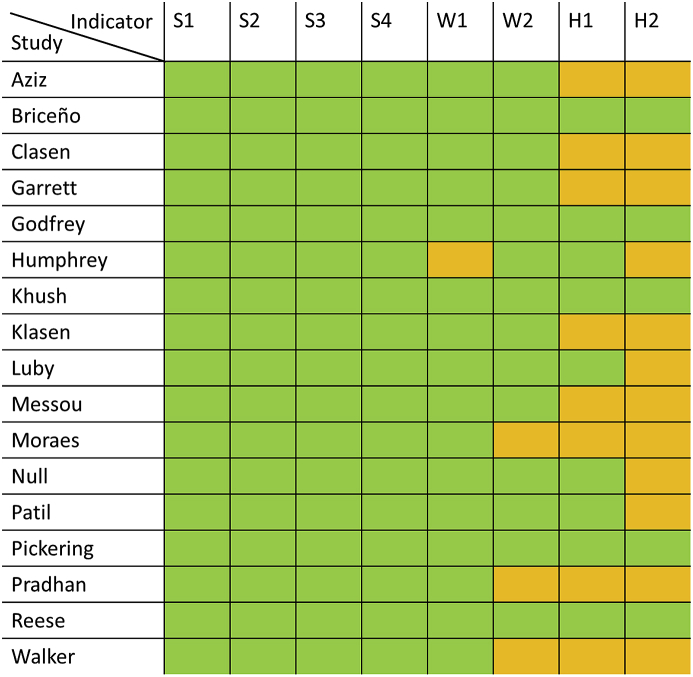
**Reporting of the eight WASH indicators in the intervention group post-intervention by sanitation study;** green: indicator reported, orange: indicator not reported, S1: open defecation/unsafe child faeces disposal, S2: basic sanitation services, S3: safely managed sanitation services, S4: community coverage with basic sanitation services, W1: basic drinking water services, W2: safely managed drinking water services, H1: basic handwashing facilities, H2: handwashing with soap after potential faecal contact. (For interpretation of the references to colour in this figure legend, the reader is referred to the Web version of this article.)

The frequency of reporting of the eight WASH indicators post-intervention in the intervention group in the 17 sanitation studies is shown in Fig. 3. While the four sanitation indicators could always be extracted from the study reports, the two hygiene indicators but also drinking water quality were more rarely reported.

#### 3.1.2 Analysis of the association between estimated faecal contamination and the effectiveness of sanitation interventions on diarrhoeal disease

Using meta-regression analysis, estimated faecal environmental contamination as represented by the score of the FAECI and the squared FAECI score were strongly associated with the relative risks of diarrhoea of intervention studies (p = 0.006). The model including all studies explained 53% of the between-study variance. Additional binary indicators of urban versus rural setting and sanitation versus combined WASH interventions, and a continuous indicator for the difference of the FAECI between intervention and control group (the “delta FAECI”) were not significantly associated with diarrhoea risk and did not change the association of the other variables in the model.

**Fig. 4:**
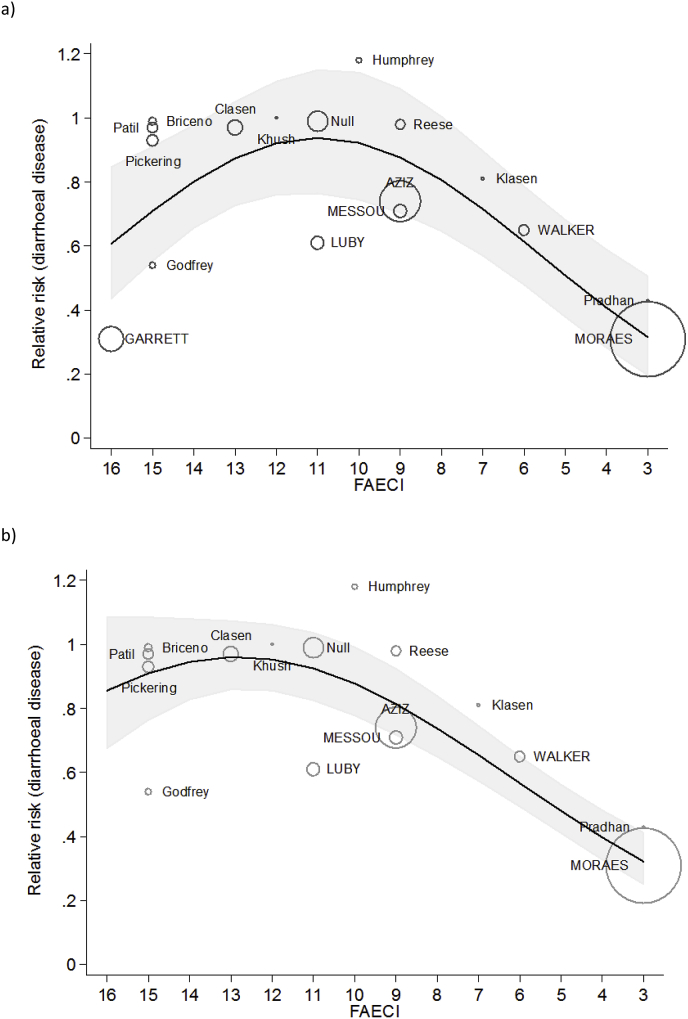
**Relative risks of diarrhoeal disease as a function of the FAECI a) including all studies, b) excluding one study identified as a potential outlier;** black line: predicted mean relative risks, shaded area: 95% confidence interval, circles represent relative risk estimates of individual studies, circle sizes are drawn proportional to the inverse of the relative risk's variance to emphasize differences in the precision of the estimates, first author name written in uppercase means significant relative risk estimates at p < 0.05, FAECI: Faecal Contamination Index.

Predicted relative risks from the meta-regression model ranged from a minimum of 0.32 (0.20, 0.51) at a FAECI of three (low estimated faecal contamination, note: as none of the included sanitation interventions yielded a FAECI score below 3, we did not predict relative risks for scores below three) to 0.94 (0.77, 1.15) at a FAECI of 11 (high estimated faecal contamination). The majority of interventions with a FAECI score between 8 and 13 did not show a significant effect of diarrhoea and hence also the predicted relative risks from the model were equivalent to no effect (i.e., confidence intervals include 1, p > 0.05). At a FAECI of 14–16 predicted relative risks declined again due to two studies that show low relative risks at a high FAECI (Fig. 4, a).

Using studentized deleted residuals, one study (Garrett et al., 2008) was identified as an outlier (Viechtbauer and Cheung, 2010). Running the meta-regression model excluding this study showed very strong associations (p < 0.0001) between the FAECI score, its squared term and the relative risk estimates of the interventions (Fig. 4, b). This model explained 88% of the between-study variance and also did not show the decline of predicted mean relative risk estimates at a large FAECI.

The delta FAECI, included separately and in combination with its squared term, was not significantly associated with the effect sizes of the interventions.

### 3.2 Estimation of country, regional and total proportions of the population from low- and middle-income countries living in communities in which basic sanitation coverage exceeds a defined threshold

#### 3.2.1 Literature search to establish health-relevant community sanitation coverage thresholds

From an initial list of 3800 citations, we selected 145 studies for abstract review and subsequently 49 studies for full text review. Of those, five studies reported the association between community coverage thresholds and diarrhoeal disease impacts (Table 4). Based on the recent systematic review and meta-analysis of intervention studies (Wolf et al., 2018a), and support from three of the other four studies (Andres et al., 2014; Jung et al., 2017b; Larsen et al., 2017), we chose 75% as a threshold for reporting as this threshold was the most frequently reported one, and 95% as a threshold indicating near complete coverage.

**Table 4:** Studies investigating the existence of community sanitation coverage thresholds for diarrhoea impacts.

citation	location	level of sanitation service provision	community coverage threshold	impact	study design
Andres et al. (2014)	India, rural	basic sanitation services	two thresholds: ∼30% (before 30% basically no change on diarrhoea) and ∼75% (around 50% of diarrhoea reduction after 75%)	47% diarrhoea reduction in children communities in a village with 100% sanitation coverage compared to children in communities with 0% coverage	cross-sectional
Harris et al. (2017)	Mali, rural	sanitation facilities (including basic, shared and unimproved)	no threshold (tested for 20%. 40% and 60% sanitation coverage), no association between increased sanitation coverage and diarrhoea	–	cross-sectional
Jung et al. (2017b)	various low-income	basic sanitation services	threshold at 60%	56% diarrhoea reduction at 100% community coverage with basic sanitation (OR 0.44 (0.29, 0.67)), 18% diarrhoea reduction < 60% coverage (OR 0.82 (0.77, 0.87))	cross-sectional (DHS surveys)
Larsen et al. (2017)	various	sanitation facilities (including improved and unimproved private facilities)	threshold between 30% and 100%	6% diarrhoea reduction (AOR 0.94 (0.91–0.97)) for children with household-level sanitation access in communities with 100% vs. 1–30% coverage	meta-analysis of survey data
Wolf et al. (2018a)	various	mainly basic sanitation services, depending on study	threshold at ≥75%	45% diarrhoea reduction in high coverage studies versus 24% in low coverage studies (five studies with ≥85% community coverage (RR 0.55 (0.34, 0.91)) versus 16 studies ≤65% coverage (RR 0.76 (0.51, 1.13)))	systematic review and meta-analysis of intervention studies

OR: odds ratio, AOR: adjusted odds ratio, RR: relative risk; basic sanitation includes improved sanitation facilities that are not shared between two or more households (WHO and UNICEF, 2017).

Furthermore, there was evidence for an association between community coverage with basic sanitation and malnutrition (Alderman et al., 2003; Cameron et al., 2017; Fuller et al., 2016; Gertler et al., 2015; Harris et al., 2017; Larsen et al., 2017; Vyas et al., 2016), infectious diseases (Fuller and Eisenberg, 2016), anaemia (Larsen et al., 2017), trachoma (Garn et al., 2018), cognitive development (Cameron et al., 2017) and child mortality (Geruso and Spears, 2015). For soil-transmitted helminths, one study found an association with community sanitation (Forrer et al., 2016), and one did not (Oswald et al., 2017).

#### 3.2.2 Data extraction from national household surveys

From the JMP data repository we identified a total of 111 countries for which survey microdata on use of basic sanitation services at PSU-level were available. The countries and territories included cover 29 out of 31 low-income, 47 out of 52 lower middle-income, 33 out of 57 upper middle-income and 2 (Trinidad and Tobago and Uruguay) out of 55 high-income countries and territories (Table 5).

**Table 5:** Number of countries and territories with available data at community-level (PSU-level) on use of basic sanitation services by region.

Region	Number of countries with community-level data on basic sanitation services	Total number of low- and middle-income countries by region	Total number of countries by region
African Region	42	46	47
Region of the Americas^a^	22	26	35
Eastern Mediterranean Region	14	15	22
European Region	16	20	53
South-East Asia Region	10	11	11
Western Pacific Region	7	20	27
Total	111	138	195

a Includes Uruguay and Trinidad and Tobago as high-income countries; basic sanitation includes improved sanitation facilities that are not shared between two or more households (WHO and UNICEF, 2017).

Surveys had been conducted between the years 1998 and 2017 (mean and median at 2012). 78% (87/111) of surveys were collected after 2010. The included countries cover 78% of the world population and 92% of the population in low- and middle-income countries. A complete list of countries included in the analysis, the survey type, year, region and income status are provided in Appendix D - Country estimates. About four hundred (401) observations (each observation represents a household) were deleted because of duplicates of households in the same PSU and country. In total, over 2 million (2,034,497) observations in a total of 93,269 PSUs were included. The mean and median number of PSUs per survey was 840 and 448 respectively and ranged from a minimum of 100 PSUs (Saint Lucia) to a maximum of 28,524 PSUs (India). The mean and median number of households surveyed per cluster was 22 and 21 respectively and ranged from a minimum of 1 household to a maximum of 764 households.

#### 3.2.3 Analysis of the population living in communities above a defined threshold of sanitation coverage

**Fig. 5:**
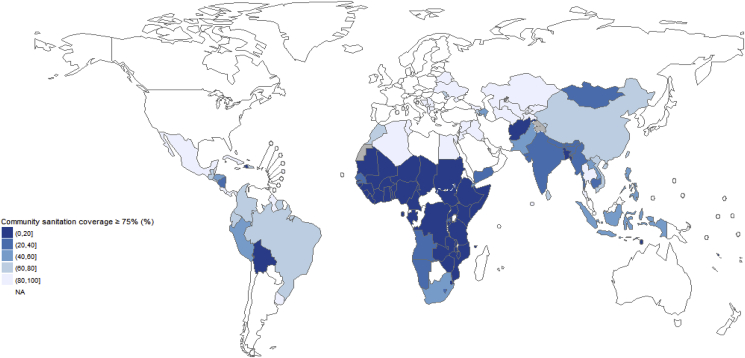
**Community sanitation coverage ≥75% by country;**“Community sanitation coverage” means the percentage of the population living in communities in which access with basic sanitation services ≥75%, basic sanitation includes improved sanitation facilities that are not shared between two or more households (WHO and UNICEF, 2017).

The percentage of the population living in communities in which more than 75% and 95% of the population are covered with basic sanitation services by country is given in Appendix D - Country estimates and is shown in Fig. 5. Regional and total aggregates are given in Table 6.

**Table 6:** Regional and total estimates of the percentage of the population from low- and middle-income countries living in communities with high sanitation coverage.

	population (percentage (95% CI)) living in communities with the given level of basic sanitation coverage	
Region	**>75%**	**>95%**
African Region	13.3% (11.1%, 16.0%)	6.2% (4.4%, 8.8%)
Region of the Americas	75.8% (73.7%, 77.7%)	46.1% (43.9%, 48.2%)
Eastern Mediterranean Region	54.8% (51.9%, 57.6%)	35.7% (32.1%, 39.6%)
European Region	93.3% (91.3%, 94.9%)	79.6% (77.4%, 81.6%)
South-East Asia Region	31.9% (30.9%, 32.9%)	12.7% (9.3%, 17.1%)
Western Pacific Region	63.2% (26.3%, 89.2%)	30.6% (5.8%, 75.8%)
Total	45.3% (34.7%, 56.4%)	23.7% (14.9%, 35.4%)

CI: confidence interval, results apply to low- and middle income countries, regions according to WHO regional groupings (WHO, 2018b), basic sanitation includes improved sanitation facilities that are not shared between two or more households (WHO and UNICEF, 2017).

## 4 Discussion

### 4.1 Main results

#### 4.1.1 Faecal environmental contamination in sanitation intervention studies and its association with the effectiveness of sanitation interventions on diarrhoeal disease

The analysis shows a non-linear association between the estimated level of faecal contamination in the community, as assessed by the FAECI, and impacts of sanitation interventions on diarrhoeal disease. It suggests that sanitation interventions are more effective at reducing diarrhoea at lower levels of faecal contamination, and that interventions are less likely to show diarrhoea reductions if faecal contamination in the community remains above a certain level.

#### 4.1.2 Estimates of the population living in communities above a defined threshold of sanitation coverage

The percentage of the population from low- and middle-income countries living in communities with more than 75% and 95% coverage with basic sanitation services is estimated at 45% and 24% respectively. Large regional discrepancies exist, with less than a third of the African and South-East Asian population living in communities covered with >75% basic sanitation services.

### 4.2 General discussion

Faecal environmental contamination was assessed combining a set of WASH indicators that cover many household- and community-level transmission pathways for diarrhoeal disease (compare to Fig. 1). Our results show that estimated faecal contamination is an important determinant of a sanitation intervention's potential to reduce diarrhoeal disease. The level of faecal environmental contamination therefore assists in interpreting differing results from WASH interventions for diarrhoeal disease reduction. The different WASH indicators on which information is collected can provide guidance on a minimum set of indicators for monitoring and reporting in all WASH research studies in order to semi-quantitatively assess the likelihood of faecal contamination after the intervention.

Some of the WASH indicators measure behaviours (S1 and H2) while another access to or presence of specific infrastructure (H1) or both depending on the study's reporting (S2, S3, S4, W1, W2). Improving use of or access to services requires different intervention strategies. Examples include health risk communication and education for improving use (Anthonj et al., 2018) and subsidy and hardware provision interventions for improving access (Garn et al., 2017). Improved data on use of services or facilities could probably improve estimates of faecal contamination.

The analysis suggests that settings with low faecal contamination post-intervention are more likely to show impacts on diarrhoeal disease than settings with high prevailing faecal contamination. In poor settings, which often carry the burden of considerable faecal contamination of the environment, sanitation and WASH interventions are crucially important, and should be considered as a necessary but possibly insufficient step for achieving disease reduction (Clasen et al., 2014). In the spirit to “leave no one behind” and get communities to a sufficiently reduced level of faecal environmental contamination, WASH interventions should continue to be made in these highly contaminated settings, to progressively reduce faecal contamination among these most vulnerable populations. The lack of impacts on diarrhoea after a WASH intervention cannot necessarily be interpreted as an intervention's ineffectiveness, a point that has already been made more than 20 years ago (VanDerslice and Briscoe, 1995). The outcome of any single intervention depends not only on whether that intervention is used sustainably but also on what other transmission pathways are important in the context of the study communities (Robb et al., 2017). Even in settings with high faecal contamination before the intervention, disease reduction may be achieved if the level of faecal contamination is reduced below a certain level. This underlines the importance of interventions that reach entire communities ensuring that everyone uses a toilet that safely contains excreta (WHO, 2018a) and that progressively reduces faecal contamination of the environment– which many interventions of the past failed to achieve (Sclar et al., 2016). WASH interventions need plans for operation and maintenance, oversight and regulation, monitoring and an accompanying enabling environment to ensure sustainability and prevent back slippage. Sanitation Safety Planning (SSP) is a local level risk assessment and management tool that can be used to identify priority risks along the sanitation chain and make improvements to technology, behaviours and management to reduce exposure. SSP also helps coordinate improvements and monitoring by multiple actors along the sanitation chain (WHO, 2015). Deleting one study (Garrett et al., 2008) identified as an outlier increased the strength of association between the FAECI and the relative risk estimates of the interventions. The excluded study might indeed be different from the other sanitation studies as it was conducted in a very sparsely populated area, where contact with community faecal contamination may have been reduced..

We had initially planned to validate the FAECI on another WASH category and attempted to extract data on the FAECI indicators from 14 drinking water filtration interventions. However, none of the filtration intervention studies reported whether sanitation facilities were safely managed, the presence of a handwashing facility with soap and water or observed handwashing with soap. While most filtration studies reported the presence of latrines or toilets it was often not clear whether these facilities were of an improved or unimproved technology. This underlines the importance of a minimum set of WASH indicators that should be reported in all WASH intervention studies independent of the WASH category addressed.

The FAECI should be regarded as a first attempt to simply, transparently and reasonably estimate the level of faecal contamination in a community environment in a way that is comparable across various intervention locations. We believe that even with slightly different indicators or different scoring, the general results and broad conclusions would be similar. We had – for example - constructed a different version of the index that included a combined measure of unsafe disposal or presence of child and animal faeces, which was however not pursued due to data scarcity on animal faeces or animal presence. Analysis of the estimated faecal contamination assessed with this modified index led to basically the same results as the here proposed one (results in Appendix A – Main, A.2). The index is semi-quantitative and does not intend to present an exact measure of faecal contamination. We caution against interpreting the size of the score too precisely and using the score of the FAECI for influencing targeting of resources. Future developments could include harmonized definitions for the proposed indicators, examine non-household settings such as schools, healthcare facilities and workplaces and explore more sophisticated approaches to estimating faecal contamination such as a latent variable approaches that treats faecal environmental contamination as an unobserved variable that can be modelled from a set of observed variables (Cai, 2012).

The FAECI includes community sanitation coverage as one indicator, which has recently been shown to have various health benefits independent from household-scale sanitation coverage (e.g., studies listed in Table 4 and (Fuller and Eisenberg, 2016; Garn et al., 2018; Jung et al., 2017a)). This paper provides the first country, regional and total estimates of the population from low- and middle-income countries living in communities with defined coverage levels of basic sanitation services that are based on nationally-representative and standardized data. It thereby completes harmonized and country-level data availability on the proposed minimum set of WASH indicators.

### 4.3 Limitations

#### 4.3.1 Faecal environmental contamination in sanitation intervention studies using the FAECI and its association with the effectiveness of sanitation interventions on diarrhoeal disease

For the proposed FAECI score, the different WASH indicators receive equal weighting though the strength of their associations with health may differ and there are likely important interactions (Kolsky and Blumenthal, 1995). Poor sanitation services will generally have a higher impact on the overall FAECI compared to poor drinking water supply or hand hygiene as four indicators relate to sanitation. This is justified as open defecation and poor sanitation can be considered the fundamental drivers of faecal contamination in a community (Daniels et al., 2016). In addition, although ingestion of unsafe water potentially increases faecal exposure, increased water availability in general protects against faecal exposure and disease (Pickering and Davis, 2012; Wang and Hunter, 2010). Some indicators are related to each other. If, for example, sanitation facilities remain mainly of an unimproved technology post-intervention, both S2 and S3 and usually also S4 will receive a high score. However, these indicators allow a differentiation between different levels of access to basic sanitation. We did not assess the applicability of the FAECI on other health outcomes (e.g. stunting), which may show different thresholds of estimated faecal contamination.

Not all indicators were reported in all included studies. Studies with the lowest estimated faecal contamination reported less information relevant for the FAECI indicators compared to the rest of the studies, which might have influenced the size of the FAECI (Fig. 3) (Moraes et al., 2003; Pradhan and Rawlings, 2002; Walker et al., 1999). None of the sanitation studies reported safe management of sanitation facilities or water sources as defined by the JMP (WHO and UNICEF, 2017), which required an adaptation of the definitions for this study (details in Table 1). No study reported observed handwashing with soap after potential faecal contact (H2). For the five studies for which information on this indicator could be extracted, we inferred low actual handwashing with soap from low self-reported handwashing or high contamination of hands. Study data on some of the indicators are likely to be subject to information bias. People under observation might wash their hands more frequently than they usually do (Hawthorne effect) and self-reported behaviour, such as on open defecation, might lead to under-reported open-defecation (McCambridge et al., 2014; Savović et al., 2012).

The FAECI does not include an exhaustive list of potential pathways for transmission of faecal pathogens (Fig. 1). Indicators are only proxy-measures, i.e., they do not provide an exact measure of faecal contamination. Due to data limitations, the indicator on handwashing with soap (H2) includes only handwashing after potential faecal contact and omits food-related handwashing occasions. Again mainly due to data limitations, food and soil contamination or contamination via flies (Bauza et al., 2018; Chavasse et al., 1999; Gautam, 2015; Morita et al., 2017) is not included. These pathways are partly dependent on the indicator (S1), which assesses presence of human faeces in the environment. Alternative ways for introducing faeces in the environment, such as wastewater irrigation (Qadir et al., 2010), are however not covered. The index also lacks information on the presence of animal faeces, which can represent an important pathway for diarrhoeal disease transmission (Ercumen et al., 2017; Penakalapati et al., 2017; Zambrano et al., 2014). The presence of animal faeces post intervention was reported by only one (Pickering et al., 2015) of the included studies. Though currently not included in the index, the presence of animal faeces should be reported in any WASH intervention study with diarrhoea or other health outcomes.

#### 4.3.2 Analysis of the association between estimated faecal contamination and the effectiveness of sanitation interventions on diarrhoeal disease

Results from meta-regression, as used in this analysis, are observational and do not establish causation between the predictor and the outcome (Thompson and Higgins, 2002). As the sample size – the number of sanitation interventions – is limited, the assessment of the association between covariates and the outcome is limited. The I^2^ statistic, a measure of inconsistency across study findings (Borenstein et al., 2009, p. 16), was high (92%), which is consistent with our hypothesis of substantial differences in background characteristics such as different levels of faecal environmental contamination but might also reflect differences in terms of intervention type and uptake, study methods, settings, and populations. Results from meta-regression suggest that estimated faecal environmental contamination can explain an important part of the variance.

#### 4.3.3 Estimates of the population living in communities above a defined threshold of sanitation coverage

The estimates are based on a limited number (n = 111) of mostly low- and middle-income countries. Furthermore, even though most surveys were conducted recently, 16 surveys data back to before 2010 (Appendix D - Country estimates). Especially for these countries it is likely that community coverage with basic sanitation services has increased by a certain extent. Use of sanitation facilities in DHS or MICS is self-reported and may therefore be biased; selection bias, e.g., from non-response, might be an additional source of bias in cross-sectional survey data (Levin, 2006).

Due to data constraints at cluster-level of national household surveys, estimates of community sanitation coverage are estimates of coverage with basic sanitation services and not necessarily safely managed services. In 2015, the JMP estimated that 68% of the world population used basic sanitation services of which only 39% were estimated to be safely managed (WHO and UNICEF, 2017). Community coverage estimates with safely managed sanitation services would therefore likely to be considerably lower than estimates for community coverage with basic sanitation services.

Not all previous evidence showed that greater community sanitation coverage leads to positive health outcomes (Harris et al., 2017; Oswald et al., 2017). One of those studies reported continuing high levels of open defecation and coverage included any sanitation facility (Harris et al., 2017). In the other study, community coverage levels with any kind of latrine as well as access to an improved drinking water source remained under 60% even in the high sanitation coverage group (Oswald et al., 2017). Higher sanitation coverage might have no impact on faecal contamination or health if facilities are not safe (Berendes et al., 2017; Daniels et al., 2016), if community coverage remains low (Odagiri et al., 2016), or if alternative pathways for transmission of faecel material exist (Yajima et al., 2009).

## 5 Conclusions

A large proportion of the world's population lives in communities that are vulnerable to significant faecal contamination from poor management of excreta. We propose a first attempt to estimate the level of faecel contamination in an intervention setting. Results of the analysis suggest that WASH interventions are more likely to lead to reductions of diarrhoeal disease when faecal contamination of the living environment has been reduced below some threshold level. This underlines the importance of interventions that reach whole communities assuring that everybody uses a safe toilet and sanitation system that separates excreta from human contact along the whole sanitation chain. WASH interventions may show no impact on diarrhoea because of persisting faecal contamination due to for example incomplete community coverage or use of sanitation facilities that do not safely contain faecal material or treat it to a level suited to the end use or disposal type. Such interventions might nevertheless be necessary interim steps to reduce faecal environmental contamination for achieving disease reduction in the future. The assessment of a minimum set of WASH indicators is useful for the evaluation of the prevailing major pathways of faecal transmission and of the intervention's effectiveness.

## Conflicts of interest

None.
