# Supplementary material for: A Faecal Contamination Index for interpreting heterogeneous diarrhoea impacts of water, sanitation and hygiene interventions and overall, regional and country estimates of community sanitation coverage with a focus on low- and middle-income countries
Source: Int J Hyg Environ Health. 2019 Mar;222(2):270–82. doi: 10.1016/j.ijheh.2018.11.005 (PMC6417992; doi:10.1016/j.ijheh.2018.11.005)
Supplement: Appendix_A [file mmc1.docx]

# Appendix A

## A.1: Confidence intervals at regional and global scale

The relation between regional and country specific standard errors is given in equation A.1

|  | $\sigma_{reg}=\sqrt{\frac{\sum_{i=1}^{n} N_{i}^{2}\sigma_{i}^{2}}{\left( \sum_{i=1}^{n} N_{i} \right)^{2}}}$ | (Eq. A.1) |
| --- | --- | --- |

, where $\sigma_{i}$ and $\sigma_{reg}$ are the standard errors on the logit scale at country*_i_* and regional level and $N_{i}$ is the population in country*_i._*

## A.2: Results of an alternative version of the Faecal Contamination Index

We had constructed a different version of the index for which S1 was open defecation, S3 was a combined measure of disposal or presence of child or animal faeces and safely managed sanitation was not included. This modified index of faecal contamination led to basically the same results as the one proposed in the main manuscript.

In meta-regression the score of this modified FAECI and its squared term were associated with the relative risks of diarrhoea of intervention studies (p=0.02). The model including all studies explained 42% of the between-study variance.

| a) |
| --- |
| 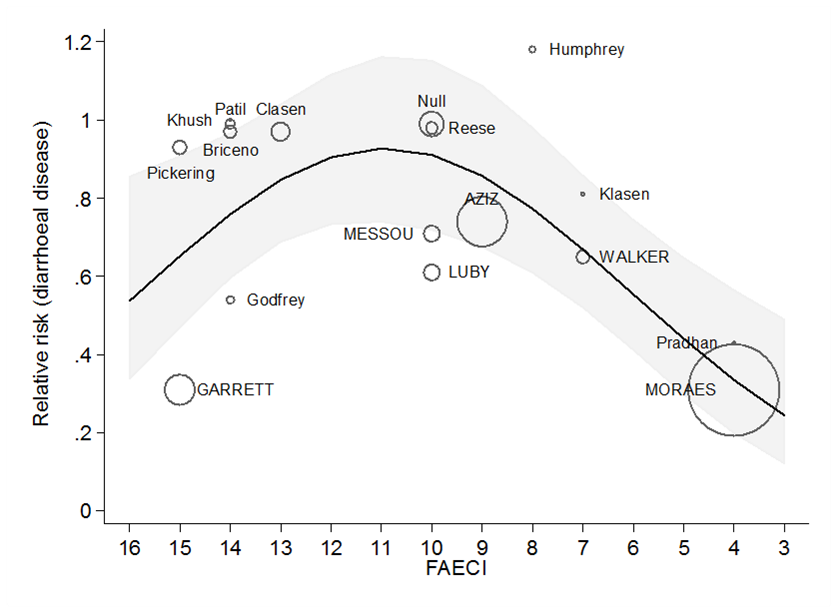 |
| b) |
| 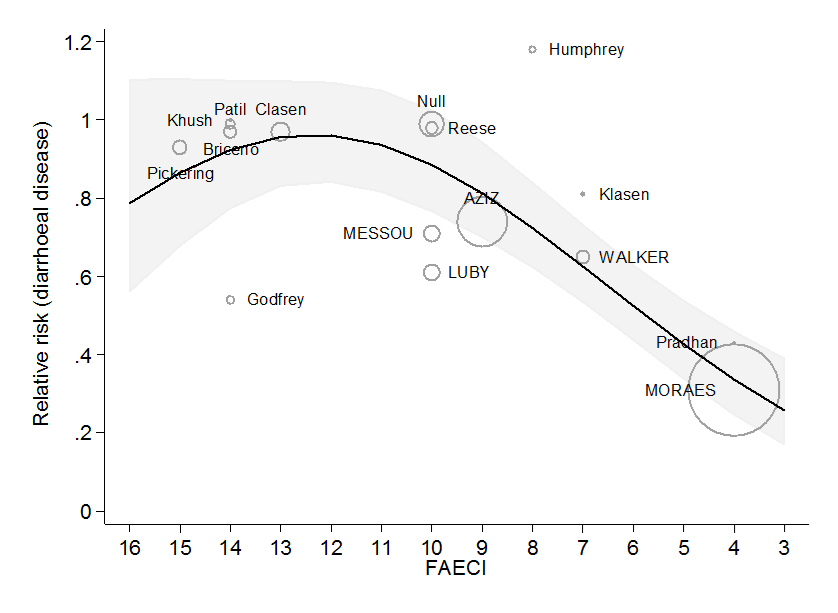 |
| Figure A.1: Relative risks of diarrhoeal disease as a function of the FAECI (modified version) a) including all studies, b) excluding one study identified as a potential outlier;  black line: predicted mean relative risks, shaded area: 95% confidence interval, circles represent relative risk estimates of individual studies, circle sizes are drawn proportional to the inverse of the relative risk’s variance to emphasize differences in the precision of the estimates, first author name written in uppercase means significant relative risk estimates at p<0.05, FAECI: Faecal Contamination Index |
