# Supplementary material for: A Faecal Contamination Index for interpreting heterogeneous diarrhoea impacts of water, sanitation and hygiene interventions and overall, regional and country estimates of community sanitation coverage with a focus on low- and middle-income countries
Source: Int J Hyg Environ Health. 2019 Mar;222(2):270–82. doi: 10.1016/j.ijheh.2018.11.005 (PMC6417992; doi:10.1016/j.ijheh.2018.11.005)
Supplement: Appendix_B [file mmc2.docx]

**
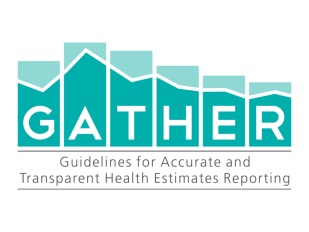
Checklist of information that should be included in new reports of global health estimates**

| Item # | Checklist item | Reported in the following section |
| --- | --- | --- |
| Objectives and funding | | |
| 1 | Define the indicator(s), populations (including age, sex, and geographic entities), and time period(s) for which estimates were made. | 🡪 methods: subtitled sections “Literature search to establish meaningful community sanitation coverage thresholds” and “Data extraction from national household surveys” |
| 2 | List the funding sources for the work. | 🡪 Acknowledgements |
| Data Inputs | | |
| *For all data inputs from multiple sources that are synthesized as part of the study:* | | |
| 3 | Describe how the data were identified and how the data were accessed. | 🡪 methods: subtitled section “Data extraction from national household surveys” |
| 4 | Specify the inclusion and exclusion criteria. Identify all ad-hoc exclusions. | 🡪 methods: subtitled section “Data extraction from national household surveys” |
| 5 | Provide information on all included data sources and their main characteristics. For each data source used, report reference information or contact name/institution, population represented, data collection method, year(s) of data collection, sex and age range, diagnostic criteria or measurement method, and sample size, as relevant. | 🡪 methods: subtitled section “Data extraction from national household surveys” |
| 6 | Identify and describe any categories of input data that have potentially important biases (e.g., based on characteristics listed in item 5). | NA (input data are from standardized, country-representative observations) |
| *For data inputs that contribute to the analysis but were not synthesized as part of the study:* | | |
| 7 | Describe and give sources for any other data inputs. | NA (all data from national household surveys) |
| *For all data inputs:* | | |
| 8 | Provide all data inputs in a file format from which data can be efficiently extracted (e.g., a spreadsheet rather than a PDF), including all relevant meta-data listed in item 5. For any data inputs that cannot be shared because of ethical or legal reasons, such as third-party ownership, provide a contact name or the name of the institution that retains the right to the data. | https://dhsprogram.com/data/;  http://mics.unicef.org/surveys;  http://nicaragua.unfpa.org/es/publicaciones/encuesta-nicarag%C3%BCense-de-demograf%C3%ADa-y-salud-endesa-2011-%E2%80%93-2012-informe-final; http://www.ecuadorencifras.gob.ec/enemdu-2016/; https://ww2.ibge.gov.br/english/estatistica/populacao/trabalhoerendimento/pnad2013/default.shtm; http://www.who.int/healthinfo/sage/en/; https://datacatalog.worldbank.org/dataset/sri-lanka-world-health-survey-2003 |
| Data analysis | | |
| 9 | Provide a conceptual overview of the data analysis method. A diagram may be helpful. | not provided as only means calculated by cluster and country |
| 10 | Provide a detailed description of all steps of the analysis, including mathematical formulae. This description should cover, as relevant, data cleaning, data pre-processing, data adjustments and weighting of data sources, and mathematical or statistical model(s). | 🡪 methods: subtitled sections “Data extraction from national household surveys” and subtitled section “Analysis of the population: living in communities above a defined threshold of sanitation coverage” |
| 11 | Describe how candidate models were evaluated and how the final model(s) were selected. | NA (only means calculated by cluster and by country) |
| 12 | Provide the results of an evaluation of model performance, if done, as well as the results of any relevant sensitivity analysis. | NA (only means calculated by cluster and by country) |
| 13 | Describe methods for calculating uncertainty of the estimates. State which sources of uncertainty were, and were not, accounted for in the uncertainty analysis. | 🡪 methods: subtitled sections “Data extraction from national household surveys” and “Analysis of the population: living in communities above a defined threshold of sanitation coverage”, 2^nd^ paragraph |
| 14 | State how analytic or statistical source code used to generate estimates can be accessed. | 🡪 methods: subtitled section: Analysis of the population: living in communities above a defined threshold of sanitation coverage, last paragraph |
| Results and Discussion | | |
| 15 | Provide published estimates in a file format from which data can be efficiently extracted. | 🡪 results: Table 6 (regional and total aggregates), Appendix D (country estimates) |
| 16 | Report a quantitative measure of the uncertainty of the estimates (e.g. uncertainty intervals). | 🡪 results: Table 6 (regional and total aggregates), Appendix D (country estimates) |
| 17 | Interpret results in light of existing evidence. If updating a previous set of estimates, describe the reasons for changes in estimates. | 🡪discussion: subtitled sections: “General discussion”, last paragraph and “Limitations”: ”Estimates of the population living in communities above a defined threshold of sanitation coverage”, last paragraph |
| 18 | Discuss limitations of the estimates. Include a discussion of any modelling assumptions or data limitations that affect interpretation of the estimates. | 🡪 discussion: subtitled section: “Limitations”: ”Estimates of the population living in communities above a defined threshold of sanitation coverage” |

*This checklist should be used in conjunction with the GATHER statement and Explanation and Elaboration document, found on gather-statement.org*
